# The Use of Cannabidiol in Patients With Low Back Pain Caused by Lumbar Spinal Stenosis: An Observational Study

**DOI:** 10.7759/cureus.29196

**Published:** 2022-09-15

**Authors:** Brock K Bakewell, Matthew Sherman, Kimberly Binsfeld, Asif M Ilyas, Stephen A Stache, Saloni Sharma, David Stolzenberg, Ari Greis

**Affiliations:** 1 Foundation for Opioid Research & Education, Rothman Orthopaedic Institute, Philadelphia, USA; 2 Orthopaedics and Rehabilitation, Rothman Orthopaedic Institute at Thomas Jefferson University, Philadelphia, USA; 3 Department of Medical Cannabis, Lambert Center at Thomas Jefferson University, Philadelphia, USA

**Keywords:** quality of life, pain management, low back pain (lbp), cannabidiol (cbd), lumbar spinal canal stenosis

## Abstract

Background

Spinal stenosis is a degenerative narrowing of the spinal canal with encroachment on the neural structures by surrounding bone and soft tissue. This chronic low back condition can cause restrictions in mobility, impairment of daily activities, opioid dependence, anxiety, depression, and reduced quality of life. Spinal stenosis can be treated through surgical and nonsurgical methods, but neither has proven consistently reliable. Cannabidiol (CBD) has also been observed to have anxiolytic, anti-inflammatory, antiemetic, and antipsychotic behaviors. CBD may provide greater nonsurgical treatment options for the pain associated with spinal stenosis while minimizing the need for opioids. An observational study was undertaken to assess the effects of CBD on patients suffering from chronic spinal stenosis.

Methodology

This observational study was investigator-initiated and designed to determine the effect of hemp-derived CBD gel caps for patients with spinal stenosis related to low back pain and leg pain relative to patient outcomes, medication utilization, and quality of life outcome measures. A total of six physician visits would be required where a set of surveys would be filled out each four weeks apart.

Results

The study population consisted of 48 patients. The patient population’s age ranged from 63 to 95 years and was normally distributed, with a mean age of 75 ± 7.13 years. The sex distribution was 33% male and 67% female patients. The pain was broken down between the six visits for each of the following four questions: pain right now, usual pain level during the week, best pain level during the week, and worst pain level during the week. Usual pain levels (p < 0.001) and worst pain levels (p < 0.005) demonstrated statistically significant improvement over time, while pain right now (p > 0.05) and best pain level (p > 0.05) stayed consistent throughout without statistical significance.

Conclusions

This open-label, prospective, observational study found that treatment with hemp-derived CBD gel caps was associated with significant improvements in pain scores and several quality-of-life measures for patients with lumbar spinal stenosis.

## Introduction

Low back pain is the leading cause of disability in 160 countries and is one of the most common health problems among individuals seeking medical care [1]. Low back pain can stem from either mechanical or unspecific origins; most cases fall into the nonspecific category where no precise cause is identified [2]. The burden of lower back pain on a global scale is extremely large. The management of the condition is largely focused on pain control through physical therapy and medications [2].

Cannabidiol (CBD) is the second most prevalent component of cannabis (marijuana). The most prevalent psychoactive component of cannabis is Δ9‐tetrahydrocannabinol (THC) [3]. THC exhibits psychoactive properties targeting the CB1 receptors of the endocannabinoid system [4], with the potential for intoxication at certain dosages. Unlike THC, CBD is nonintoxicating and has been seen to have few side effects making it safe at high doses [5]. CBD has also been observed to have anxiolytic, anti-inflammatory, antiemetic, and antipsychotic behaviors [6]. While generally used in tandem, THC and CBD have been shown to have the ability to reduce pain through several pathways such as modulation of rostral ventromedial medulla neuronal activity, antinociceptive effects in descending pain pathways, and anti-inflammatory properties by acting through prostaglandin synthesis inhibition [7]. However, THC can have unwanted side effects such as disconnected thoughts, panic attacks, altered perception, delusions, and hallucinatory experiences [8]. Without the potential for intoxication, CBD is being explored as a treatment for pain relief without side effects.

Lower back pain has a litany of causes including intervertebral disk degeneration or extrusion, Modic changes, coronavirus disease, and spondylolysis [2,9]. Spinal stenosis is the degenerative narrowing of the spinal canal with encroachment on the neural structures by surrounding bone and soft tissue. Patients diagnosed with spinal stenosis typically present with low back pain that radiates to the legs when standing and walking and improves with sitting [10]. Spinal stenosis is the most common diagnosis that leads to lumbar spine surgery in individuals over 65 [11]. This chronic low back condition can cause restrictions in mobility, impairment of daily activities, opioid dependence, anxiety, depression, and reduced quality of life [12]. Spinal stenosis can be treated through surgical and nonsurgical methods. The surgical method used to treat spinal stenosis is decompressive laminectomy with or without spinal fusion, while nonsurgical methods include physical therapy, epidural steroid injections, anti-inflammatory drugs, neuropathic pain medications, and opioid analgesics [13]. However, a recent meta-analysis found no difference in pain and physical function between surgical and nonsurgical groups in the management of spinal stenosis after four years [13]. Another study found that surgery produced better clinical outcomes in spinal stenosis patients, but ultimately nonsurgical modalities were preferred as first-line treatment because they minimized healthcare costs and prevented complications [14]. To provide greater nonsurgical treatment options for the pain associated with spinal stenosis, while minimizing the need for opioids, an observational study was undertaken to assess the effects of CBD on patients suffering from chronic spinal stenosis.

## Materials and methods

Study design

This observational study was investigator-initiated and designed to determine the effect of hemp-derived CBD gel caps for patients with spinal stenosis-related low back pain and leg pain relative to patient outcomes, medication utilization, and quality of life outcome measures. The Institutional Review Board at Thomas Jefferson University approved a standardized protocol.

Patient population

Inclusion criteria included proficiency in the English language, age greater than or equal to 60, a history of neurogenic claudication or radicular leg symptoms for greater than three months, and confirmatory radiographic imaging showing lumbar spinal stenosis at one or more levels. Patients with unstable degenerative spondylolisthesis were excluded from this study. Patients were also excluded if they had undergone any prior lumbar spinal surgeries. Patients were not excluded for any pulmonary, cardiovascular, or neurological diseases. Eligible patients were referred to a Research Assistant who would go over the informed consent highlighting both the benefits and risks. The Research Assistant would then offer the patients who enrolled compensation of $25 upon completing the first survey and a coupon for a bottle of hemp-derived CBD gel caps for the following five visits.

Study interventions

A total of six physician visits would be required where a set of surveys would be filled out each four weeks apart. The first survey would be compensated $25, and each following visit would be compensated with a coupon for a free bottle of “CBD-rich” gel caps (Ananda Hemp, Cynthiana, KY, USA). The gel caps used specifically were the 15 mg “Full Spectrum Hemp Extract Soft Gels,” containing 0.3% THC, with the packaging recommending its use as one capsule twice daily.

Study measures

Seven surveys used at each visit: Pain Numeric Rating Scale, The Roland-Morris Low Back Pain and Disability Questionnaire, The Inventory of Depression and Anxiety Symptoms Second Version (IDAS-II), Brief Inventory of Psychosocial Functioning (B-IPF), Pittsburgh Sleep Quality Index (PSQI), The Daily sessions, Frequency, Age of Onset, and Quantity of Cannabis Use Inventory (DFAQ-CU), and a Medical and Psychiatric Treatment Receipt.

Statistical analysis

Descriptive statistics were used to summarize the data collected over each of the six visits. The data were analyzed both by patient visit intervals as well as continuous data, presented as mean (standard deviation), and categorical data are presented as cell count (percentage of the total count). Analysis of variance (ANOVA) testing was performed to calculate the p-values for continuous data and chi-square testing was used for categorical data. Following the descriptive tables, a set of regressions are also presented. Each regression focused on the four different pain scores as the main dependent outcome. Significance was determined at a p-value of <0.05. All statistical analyses were done using R Studio (Version 3.6.3, Vienna, Austria).

## Results

**Figure 1:**
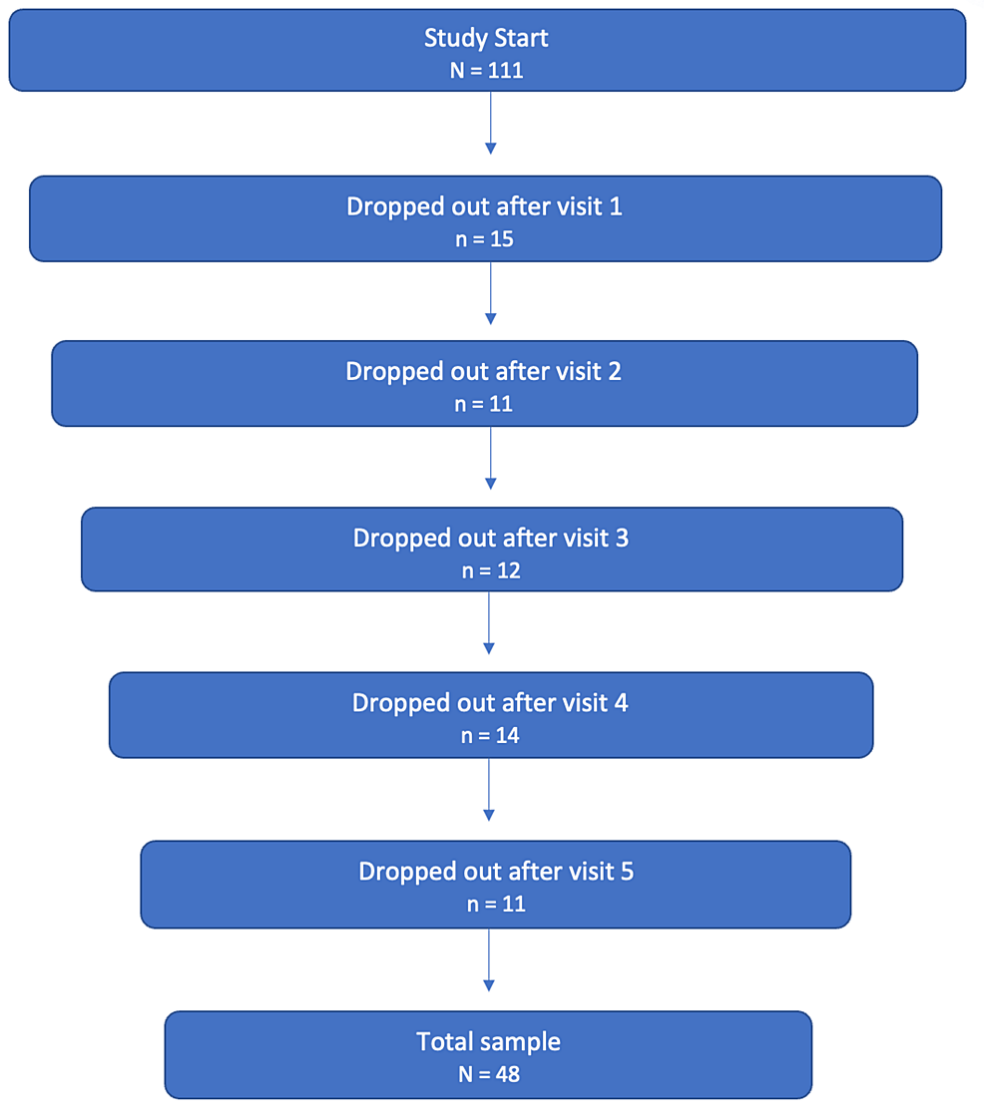
Patient dropouts per visit and the final sample count. N = initial number of participants/final sample count; n = number of patients who dropped out

During this study, a total of 111 patients provided informed consent and completed baseline questionnaires. However, participants dropped out after each visit. Only patients who completed all six visits were included in the final study sample, which resulted in a final study population of 48 patients (43% of the total enrolled) (Figure 1). The age of the patient population ranged from 63 to 95 years and was normally distributed, with a mean age of 75 ± 7.13 years. The sex distribution was 33% male and 67% female patients.

Table 1 summarizes the four pain variables. Pain was broken down between the six visits for each of the following four questions: pain right now, usual pain level during the week, best pain level during the week, and worst pain level during the week. Usual pain levels (p < 0.001) and worst pain levels (p < 0.005) demonstrated statistically significant improvement over time, while pain right now (p > 0.05) and best pain level (p > 0.05) stayed consistent throughout without statistical significance.

**Table 1:** Pain scores.

Variable	Visit 1, N = 48	Visit 2, N = 48	Visit 3, N = 48	Visit 4, N = 48	Visit 5, N = 48	Visit 6, N = 48	Overall p-value
How would you rate your pain right now	4.58 (2.28)	4.58 (2.38)	3.79 (2.50)	4.17 (2.79)	4.02 (2.61)	3.77 (2.60)	0.421
How would you rate your usual level of pain during the last week	6.02 (1.95)	5.62 (2.24)	4.60 (2.35)	4.38 (2.38)	4.52 (2.29)	4.26 (2.41)	<0.001
How would you rate your best level of pain during the last week	3.12 (1.99)	3.21 (2.27)	2.56 (2.05)	2.44 (2.23)	2.71 (2.32)	2.29 (2.16)	0.223
How would you rate you worst level of pain during the last week	7.54 (2.07)	7.31 (2.28)	6.33 (2.91)	6.17 (2.93)	6.15 (2.74)	5.75 (2.82)	0.004

Table 2 contains the questions pertaining to the Roland-Morris Low Back Pain and Disability Questionnaire, a self-reported questionnaire about how low back pain affects functional activities. This scale is graded on a 24-point scale, with each question being worth one point. Scores can range from 0 (no disability) to 24 (severe disability). At baseline, the average patient score was 10.13 ± 5.37, and after the six-month study period, the average patient score was 8.04 ± 5.62. One individual variable that reached statistical significance over the course of the six study visits was patients reported less sleep disturbances because of their back pain (p = 0.005).

**Table 2:** Roland-Morris Low Back Pain and Disability questionnaire.

Variable	Visit 1, N = 48	Visit 2, N = 48	Visit 3, N = 48	Visit 4, N = 48	Visit 5, N = 48	Visit 6, N = 48	Overall p-value
I stay at home most of the time because of my back	9 (18.8%)	5 (10.4%)	5 (10.4%)	5 (10.4%)	4 (8.33%)	4 (8.33%)	0.600
I change position frequently to try to get my back comfortable	30 (62.5%)	26 (54.2%)	26 (54.2%)	22 (45.8%)	22 (45.8%)	23 (47.9%)	0.539
I walk more slowly than usual because of my back	39 (81.2%)	36 (75.0%)	33 (68.8%)	34 (70.8%)	30 (62.5%)	31 (64.6%)	0.359
Because of my back, I am not doing any jobs that I usually do around the house	14 (29.2%)	12 (25.0%)	14 (29.2%)	8 (16.7%)	12 (25.0%)	12 (25.0%)	0.751
Because of my back, I use a handrail to get upstairs	32 (66.7%)	31 (64.6%)	30 (62.5%)	31 (64.6%)	32 (66.7%)	28 (58.3%)	0.961
Because of my back, I lie down to rest more often	22 (45.8%)	21 (43.8%)	16 (33.3%)	19 (39.6%)	16 (33.3%)	17 (35.4%)	0.708
Because of my back, I have to hold on to something to get out of an easy chair	19 (39.6%)	18 (37.5%)	17 (35.4%)	18 (37.5%)	18 (37.5%)	18 (37.5%)	0.999
Because of my back, I try to get other people to do things for me	11 (22.9%)	14 (29.2%)	8 (16.7%)	12 (25.0%)	11 (22.9%)	10 (20.8%)	0.798
I get dressed more slowly than usual because of my back	20 (41.7%)	26 (54.2%)	20 (41.7%)	16 (33.3%)	24 (50.0%)	19 (39.6%)	0.358
I only stand up for short periods of time because of my back	27 (56.2%)	23 (47.9%)	22 (45.8%)	22 (45.8%)	25 (52.1%)	22 (45.8%)	0.877
Because of my back, I try not to bend or kneel down	25 (52.1%)	22 (45.8%)	18 (37.5%)	21 (43.8%)	16 (33.3%)	23 (47.9%)	0.460
I find it difficult to get out of a chair because of my back	19 (39.6%)	16 (33.3%)	13 (27.1%)	12 (25.0%)	17 (35.4%)	15 (31.2%)	0.670
My back is painful almost all of the time	22 (45.8%)	18 (37.5%)	12 (25.0%)	16 (33.3%)	11 (22.9%)	13 (27.1%)	0.137
I find it difficult to turn over on bed because of my back	21 (43.8%)	20 (41.7%)	15 (31.2%)	12 (25.0%)	11 (22.9%)	15 (31.2%)	0.162
My appetite is not very good because of my back	1 (2.08%)	3 (6.25%)	2 (4.17%)	3 (6.25%)	0 (0.00%)	0 (0.00%)	0.262
I have trouble putting on my sock (or stockings) because of the pain in my back	21 (43.8%)	19 (39.6%)	17 (35.4%)	14 (29.2%)	14 (29.2%)	18 (37.5%)	0.620
I can only walk short distances because of my back pain	34 (70.8%)	31 (64.6%)	29 (60.4%)	25 (52.1%)	30 (62.5%)	27 (56.2%)	0.504
I sleep less well because of my back	25 (52.1%)	20 (41.7%)	13 (27.1%)	11 (22.9%)	13 (27.1%)	10 (20.8%)	0.005
Because of my back pain, I get dressed with the help of someone else	2 (4.17%)	2 (4.17%)	1 (2.08%)	1 (2.08%)	1 (2.08%)	1 (2.08%)	1.000
I sit down for most of the day because of my back	13 (27.1%)	16 (33.3%)	14 (29.2%)	11 (22.9%)	12 (25.0%)	13 (27.1%)	0.907
I avoid heavy jobs around the house because of my back	35 (72.9%)	35 (72.9%)	31 (64.6%)	34 (70.8%)	34 (70.8%)	33 (68.8%)	0.952
Because of back pain, I am more irritable and bad tempered with people than usual	12 (25.0%)	11 (22.9%)	4 (8.33%)	4 (8.33%)	6 (12.5%)	6 (12.5%)	0.076
Because of my back, I go upstairs more slowly than usual	32 (66.7%)	37 (77.1%)	31 (64.6%)	25 (52.1%)	30 (62.5%)	28 (58.3%)	0.197
I stay in bed most of the time because of my back	1 (2.08%)	0 (0.00%)	0 (0.00%)	0 (0.00%)	1 (2.08%)	0 (0.00%)	1.000

Tables 3-6 present the IDAS-II findings. The IDAS-II is a self-reported questionnaire containing 18 factor-analytically derived scales, each assessing a specific symptom of internalizing disorders, including depression, anxiety disorders, obsessive-compulsive disorder (OCD), bipolar disorder, and posttraumatic stress disorder (PTSD). Over the course of the six visits, several sleep variables were observed to improve. Improvement was seen as soon as visit three, four weeks after starting CBD. Several of these variables trended toward significance, while some reached statistical significance. Patients reported maintaining normal sleep habits (p < 0.05), feeling less exhausted (p < 0.005), had less trouble falling asleep (p < 0.05), woke up at a normal hour (p = 0.005), woke up less during the night (p < 0.05), reported sleeping better (p < 0.05), and were able to concentrate better (p < 0.05). There were no observed changes in anxiety or depression scores.

**Table 3:** Inventory of Depression and Anxiety Symptoms Second Version (IDAS-II).

Variable	Visit 1, N = 48	Visit 2, N = 48	Visit 3, N = 48	Visit 4, N = 48	Visit 5, N = 48	Visit 6, N = 48	Overall p-value
I did not have much of an appetite	1.36 (0.76)	1.49 (0.78)	1.42 (0.77)	1.50 (0.84)	1.35 (0.64)	1.26 (0.53)	0.580
I had little interest in my usual hobbies and activities	1.85 (1.12)	1.64 (0.79)	1.70 (0.91)	1.50 (0.82)	1.50 (0.80)	1.52 (0.86)	0.329
I felt optimistic	2.88 (1.14)	3.04 (0.98)	3.12 (0.96)	2.96 (1.15)	3.12 (1.08)	3.30 (0.95)	0.453
I slept less than usual	2.46 (1.30)	2.23 (1.28)	1.88 (1.00)	1.80 (0.88)	1.98 (0.93)	1.74 (0.97)	0.009
I felt fidgety, restless	2.02 (1.24)	1.66 (1.05)	1.68 (1.00)	1.51 (0.89)	1.50 (0.83)	1.66 (0.96)	0.146
I felt exhausted	2.39 (1.08)	2.41 (1.15)	2.04 (0.91)	1.77 (0.91)	1.83 (0.81)	1.93 (0.88)	0.002
I felt a pain in my chest	1.09 (0.35)	1.15 (0.51)	1.06 (0.25)	1.07 (0.33)	1.09 (0.35)	1.13 (0.50)	0.859
I felt depressed	1.64 (0.97)	1.62 (1.01)	1.42 (0.77)	1.41 (0.91)	1.56 (0.97)	1.36 (0.65)	0.516
I had trouble making up my mind	1.41 (0.78)	1.57 (0.85)	1.38 (0.71)	1.33 (0.63)	1.33 (0.75)	1.28 (0.58)	0.449
I was proud of myself	3.17 (1.28)	3.02 (1.04)	3.30 (1.02)	3.04 (1.06)	2.98 (1.19)	3.10 (1.10)	0.770
I had trouble falling asleep	2.17 (1.32)	2.17 (1.33)	1.81 (0.96)	1.64 (0.83)	1.77 (0.90)	1.67 (0.73)	0.035
I was furious	1.26 (0.61)	1.26 (0.68)	1.27 (0.64)	1.17 (0.44)	1.19 (0.45)	1.30 (0.59)	0.872
I had thoughts of suicide	1.04 (0.21)	1.00 (0.00)	1.00 (0.00)	1.02 (0.15)	1.00 (0.00)	1.02 (0.15)	0.395
I had disturbing thoughts of something bad that happened to me	1.09 (0.35)	1.06 (0.32)	1.12 (0.44)	1.09 (0.28)	1.19 (0.45)	1.04 (0.21)	0.431
I felt self-conscious knowing that others were watching me	1.34 (0.84)	1.26 (0.57)	1.21 (0.58)	1.20 (0.59)	1.27 (0.57)	1.09 (0.28)	0.450
I felt dizzy or lightheaded	1.30 (0.59)	1.23 (0.63)	1.23 (0.47)	1.20 (0.58)	1.15 (0.41)	1.13 (0.40)	0.653
I woke up early and could not get back to sleep	2.23 (1.22)	2.06 (1.17)	1.88 (1.08)	1.77 (0.89)	1.77 (0.81)	1.67 (0.92)	0.078
I was worried about embarrassing myself socially	1.30 (0.79)	1.26 (0.61)	1.35 (0.73)	1.20 (0.50)	1.21 (0.59)	1.13 (0.34)	0.545
I thought a lot about food	1.81 (0.95)	1.72 (0.97)	1.60 (0.84)	1.77 (0.99)	1.60 (0.88)	1.61 (0.93)	0.787
I became anxious in a crowded public setting	1.41 (0.75)	1.32 (0.63)	1.19 (0.57)	1.22 (0.70)	1.27 (0.64)	1.13 (0.40)	0.320
I blamed myself for things	1.48 (0.86)	1.51 (0.75)	1.34 (0.67)	1.20 (0.54)	1.33 (0.75)	1.24 (0.57)	0.193
I cut or burned myself on purpose	1.00 (0.00)	1.00 (0.00)	1.00 (0.00)	1.00 (0.00)	1.00 (0.00)	1.00 (0.00)	0.421
I felt that I had accomplished a lot	3.33 (1.12)	2.89 (0.94)	3.08 (0.96)	3.02 (1.05)	2.85 (1.09)	3.04 (1.21)	0.332
I ate when I wasn't hungry	1.83 (1.08)	1.77 (0.87)	1.65 (0.79)	1.74 (0.83)	1.71 (0.87)	1.64 (0.74)	0.908

**Table 4:** Inventory of Depression and Anxiety Symptoms Second Version (IDAS-II).

Variable	Visit 1, N = 48	Visit 2, N = 48	Visit 3, N = 48	Visit 4, N = 48	Visit 5, N = 48	Visit 6, N = 48	Overall p-value
I woke up much earlier than usual	2.20 (1.26)	2.00 (1.23)	1.56 (0.92)	1.61 (0.74)	1.69 (0.83)	1.55 (0.80)	0.005
I felt like eating less than usual	1.43 (0.83)	1.66 (0.92)	1.38 (0.77)	1.39 (0.68)	1.38 (0.64)	1.24 (0.48)	0.168
I looked forward to things with enjoyment	3.73 (1.01)	3.30 (1.11)	3.15 (1.04)	3.33 (1.08)	3.17 (1.10)	3.28 (1.10)	0.116
I had nightmares that reminded me of something bad that happened	1.22 (0.51)	1.26 (0.65)	1.21 (0.55)	1.20 (0.50)	1.23 (0.66)	1.13 (0.50)	0.927
I slept more than usual	1.48 (0.84)	1.57 (0.80)	1.56 (0.90)	1.38 (0.68)	1.35 (0.60)	1.48 (0.78)	0.658
It took a lot of effort for me to get going	1.94 (1.01)	1.89 (0.98)	1.79 (0.85)	1.91 (0.96)	1.73 (0.76)	1.70 (0.73)	0.690
I felt inadequate	1.52 (0.91)	1.40 (0.85)	1.33 (0.69)	1.43 (0.78)	1.35 (0.79)	1.21 (0.51)	0.504
I was trembling or shaking	1.07 (0.25)	1.09 (0.35)	1.12 (0.49)	1.07 (0.33)	1.04 (0.20)	1.00 (0.00)	0.518
I thought that the world would be better off without me	1.07 (0.25)	1.02 (0.15)	1.02 (0.14)	1.07 (0.25)	1.02 (0.15)	1.00 (0.00)	0.382
I had memories of something scary that happened	1.15 (0.42)	1.21 (0.59)	1.15 (0.36)	1.16 (0.42)	1.13 (0.34)	1.09 (0.36)	0.833
I felt like breaking things	1.09 (0.36)	1.09 (0.35)	1.04 (0.20)	1.07 (0.33)	1.02 (0.14)	1.04 (0.21)	0.819
I woke up frequently during the night	2.34 (1.27)	1.98 (1.23)	1.81 (0.89)	1.72 (0.81)	1.77 (0.88)	1.81 (0.99)	0.040
I felt enraged	1.13 (0.34)	1.19 (0.58)	1.06 (0.25)	1.04 (0.21)	1.13 (0.34)	1.11 (0.38)	0.443
I hurt myself purposely	1.00 (0.00)	1.02 (0.15)	1.00 (0.00)	1.09 (0.59)	1.00 (0.00)	1.00 (0.00)	0.467
I felt faint	1.15 (0.36)	1.04 (0.20)	1.04 (0.21)	1.00 (0.00)	1.04 (0.21)	1.05 (0.21)	0.044
I felt discouraged about things	1.87 (0.97)	1.79 (0.81)	1.56 (0.74)	1.66 (0.81)	1.69 (0.88)	1.61 (0.71)	0.470
I found it difficult to make eye contact with people	1.04 (0.21)	1.07 (0.33)	1.10 (0.47)	1.11 (0.44)	1.09 (0.28)	1.07 (0.25)	0.938
I got upset thinking about something bad that happened	1.43 (0.77)	1.30 (0.72)	1.17 (0.43)	1.33 (0.67)	1.17 (0.43)	1.17 (0.57)	0.215
I had trouble waking up in the morning	1.34 (0.67)	1.36 (0.64)	1.33 (0.63)	1.40 (0.78)	1.42 (0.74)	1.37 (0.77)	0.992
I lost my temper and yelled at people	1.32 (0.78)	1.23 (0.56)	1.17 (0.52)	1.15 (0.47)	1.12 (0.33)	1.20 (0.55)	0.582
My heart was racing or pounding	1.11 (0.31)	1.06 (0.25)	1.08 (0.35)	1.04 (0.21)	1.09 (0.35)	1.07 (0.33)	0.945
I thought about my own death	1.11 (0.37)	1.07 (0.25)	1.04 (0.20)	1.11 (0.38)	1.08 (0.28)	1.05 (0.21)	0.802
I found it difficult to talk with people I did not know well	1.19 (0.50)	1.15 (0.42)	1.15 (0.41)	1.16 (0.47)	1.19 (0.49)	1.13 (0.34)	0.982
I found myself worrying all the time	1.57 (0.90)	1.57 (1.02)	1.43 (0.77)	1.57 (0.86)	1.44 (0.87)	1.42 (0.89)	0.873

**Table 5:** Inventory of Depression and Anxiety Symptoms Second Version (IDAS-II).

Variable	Visit 1, N = 48	Visit 2, N = 48	Visit 3, N = 48	Visit 4, N = 48	Visit 5, N = 48	Visit 6, N = 48	Overall p-value
I had a very dry mouth	1.46 (0.82)	1.21 (0.51)	1.46 (0.62)	1.30 (0.62)	1.19 (0.45)	1.20 (0.55)	0.069
I felt hopeful about the future	3.17 (1.06)	2.98 (1.14)	2.92 (1.09)	3.04 (0.98)	2.77 (1.02)	2.87 (1.10)	0.544
I slept very poorly	2.28 (1.39)	2.10 (1.19)	1.94 (0.89)	1.64 (0.80)	1.71 (0.85)	1.66 (1.03)	0.013
I thought about hurting myself	1.11 (0.60)	1.02 (0.15)	1.00 (0.00)	1.00 (0.00)	1.00 (0.00)	1.02 (0.15)	0.314
I felt that I had a lot to look forward to	3.36 (1.13)	3.11 (1.32)	3.19 (1.02)	3.21 (1.04)	2.96 (1.22)	3.13 (1.15)	0.698
I felt much worse in the morning than later in the day	1.79 (1.27)	1.96 (1.13)	1.65 (0.96)	1.53 (1.04)	1.52 (0.92)	1.46 (0.89)	0.165
I felt drowsy, sleepy	1.62 (0.71)	1.87 (0.95)	1.74 (0.77)	1.78 (1.00)	1.54 (0.80)	1.58 (0.81)	0.358
I was short of breath	1.23 (0.67)	1.06 (0.25)	1.15 (0.41)	1.26 (0.77)	1.11 (0.38)	1.13 (0.54)	0.458
I talked more slowly than usual	1.13 (0.54)	1.06 (0.25)	1.15 (0.41)	1.09 (0.35)	1.09 (0.28)	1.04 (0.21)	0.744
I felt like I was choking	1.06 (0.32)	1.06 (0.32)	1.00 (0.00)	1.02 (0.15)	1.00 (0.00)	1.00 (0.00)	0.314
I felt like I had a lot of interesting things to do	3.23 (1.29)	2.96 (1.25)	3.10 (0.93)	2.96 (1.19)	2.96 (1.13)	3.04 (1.08)	0.818
I did not feel much like eating	1.28 (0.74)	1.40 (0.80)	1.40 (0.74)	1.31 (0.63)	1.29 (0.59)	1.27 (0.59)	0.881
I had trouble concentrating	1.81 (0.92)	1.83 (0.90)	1.54 (0.71)	1.47 (0.69)	1.46 (0.58)	1.44 (0.69)	0.027
Little things made me mad	1.53 (0.93)	1.45 (0.77)	1.25 (0.53)	1.26 (0.53)	1.42 (0.74)	1.28 (0.66)	0.274
I ate more than usual	1.52 (0.94)	1.49 (0.80)	1.60 (0.74)	1.41 (0.83)	1.49 (0.78)	1.49 (0.69)	0.922
I felt like I had a lot of energy	2.43 (1.08)	2.40 (1.06)	2.33 (1.10)	2.62 (1.17)	2.27 (1.03)	2.43 (1.09)	0.735
I rearranged things so that they were in a certain order	1.91 (1.14)	1.70 (0.91)	1.65 (0.93)	1.56 (0.89)	1.40 (0.64)	1.33 (0.76)	0.021
I washed my hands excessively	1.21 (0.62)	1.15 (0.55)	1.17 (0.43)	1.09 (0.36)	1.19 (0.57)	1.17 (0.61)	0.920
I kept racing from one activity to the next	1.26 (0.53)	1.26 (0.57)	1.15 (0.36)	1.13 (0.34)	1.15 (0.41)	1.13 (0.40)	0.516
I checked things over and over again	1.38 (0.64)	1.36 (0.61)	1.34 (0.64)	1.26 (0.53)	1.22 (0.42)	1.23 (0.52)	0.613
I felt the urge to rearrange things so that they were “just right”	1.39 (0.83)	1.40 (0.71)	1.35 (0.76)	1.30 (0.66)	1.29 (0.54)	1.18 (0.45)	0.648
I worried a lot about germs	1.17 (0.43)	1.15 (0.51)	1.21 (0.54)	1.17 (0.53)	1.12 (0.49)	1.13 (0.50)	0.971
I spoke so rapidly that others could not understand me	1.04 (0.20)	1.02 (0.15)	1.02 (0.15)	1.00 (0.00)	1.04 (0.29)	1.02 (0.15)	0.869
I felt elated for no special reason	1.22 (0.63)	1.11 (0.43)	1.21 (0.58)	1.09 (0.28)	1.09 (0.28)	1.11 (0.38)	0.518

**Table 6:** Inventory of Depression and Anxiety Symptoms Second Version (IDAS-II).

Variable	Visit 1, N = 48	Visit 2, N = 48	Visit 3, N = 48	Visit 4, N = 48	Visit 5, N = 48	Visit 6, N = 48	Overall p-value
I tried not to think about bad things from my past	1.64 (1.01)	1.51 (0.91)	1.35 (0.67)	1.52 (0.84)	1.47 (0.86)	1.26 (0.61)	0.301
I avoided small spaces	1.38 (0.97)	1.36 (0.90)	1.31 (0.85)	1.35 (0.87)	1.36 (0.90)	1.28 (0.81)	0.995
I found myself checking things, even though I knew it wasn't necessary	1.33 (0.75)	1.36 (0.67)	1.46 (0.78)	1.22 (0.51)	1.23 (0.47)	1.23 (0.56)	0.392
I avoided handling dirty things	1.21 (0.59)	1.22 (0.70)	1.12 (0.49)	1.17 (0.57)	1.15 (0.62)	1.13 (0.50)	0.952
It felt like my mind was moving “a mile a minute”	1.27 (0.58)	1.13 (0.40)	1.17 (0.43)	1.05 (0.21)	1.12 (0.33)	1.11 (0.38)	0.194
I felt like I was “on top of the world”	1.45 (0.85)	1.55 (0.93)	1.40 (0.82)	1.46 (0.84)	1.28 (0.58)	1.38 (0.83)	0.698
I avoided situations that bring up bad memories	1.44 (0.89)	1.36 (0.61)	1.19 (0.39)	1.30 (0.59)	1.21 (0.46)	1.22 (0.56)	0.264
I was afraid of getting trapped in a crowd	1.21 (0.66)	1.26 (0.71)	1.23 (0.69)	1.17 (0.68)	1.19 (0.64)	1.15 (0.47)	0.973
I felt the urge to check to make sure I had done something	1.79 (0.94)	1.62 (0.77)	1.58 (0.79)	1.72 (0.91)	1.35 (0.60)	1.41 (0.75)	0.063
I followed the same, fixed order in performing everyday tasks	1.91 (1.10)	2.00 (1.04)	1.69 (0.75)	1.48 (0.69)	1.70 (0.83)	1.67 (0.73)	0.061
My thoughts jumped rapidly from one idea to another	1.30 (0.55)	1.36 (0.67)	1.31 (0.59)	1.22 (0.47)	1.23 (0.60)	1.22 (0.47)	0.786
I felt anxious in small spaces	1.36 (0.92)	1.28 (0.77)	1.36 (0.87)	1.27 (0.75)	1.27 (0.79)	1.18 (0.65)	0.886
I felt compelled to follow certain rituals	1.40 (0.83)	1.30 (0.66)	1.21 (0.55)	1.22 (0.55)	1.25 (0.60)	1.33 (0.67)	0.699
I had difficulty touching something that was dirty	1.15 (0.62)	1.17 (0.64)	1.17 (0.64)	1.13 (0.50)	1.12 (0.61)	1.13 (0.50)	0.998
My thoughts were moving so quickly it was hard to keep up	1.13 (0.40)	1.06 (0.25)	1.09 (0.28)	1.00 (0.00)	1.06 (0.24)	1.04 (0.21)	0.283
I had so much energy it was hard for me to sit still	1.26 (0.67)	1.26 (0.79)	1.25 (0.73)	1.11 (0.38)	1.17 (0.48)	1.28 (0.75)	0.796
I tried to ignore upsetting memories	1.74 (1.24)	1.41 (0.72)	1.30 (0.62)	1.50 (0.75)	1.29 (0.65)	1.30 (0.55)	0.041
I was afraid of tunnels	1.34 (0.94)	1.30 (0.81)	1.29 (0.80)	1.27 (0.72)	1.27 (0.79)	1.22 (0.70)	0.990
I had to clean myself because I felt contaminated	1.02 (0.15)	1.00 (0.00)	1.02 (0.14)	1.00 (0.00)	1.00 (0.00)	1.04 (0.29)	0.642
I felt that I could do things that other people couldn’t	1.53 (0.93)	1.28 (0.62)	1.39 (0.74)	1.33 (0.70)	1.35 (0.70)	1.47 (0.89)	0.628
I avoided talking about bad experiences from my past	1.40 (0.77)	1.38 (0.68)	1.33 (0.60)	1.33 (0.67)	1.35 (0.67)	1.24 (0.52)	0.877
I avoided tight, enclosed spaces	1.38 (0.92)	1.32 (0.96)	1.31 (0.85)	1.33 (0.80)	1.33 (0.90)	1.24 (0.74)	0.985
I had little rituals or habits that took up a lot of my time	1.23 (0.63)	1.17 (0.43)	1.13 (0.40)	1.20 (0.50)	1.17 (0.48)	1.27 (0.69)	0.844
I avoided using public restrooms	1.15 (0.36)	1.23 (0.73)	1.19 (0.64)	1.11 (0.38)	1.17 (0.63)	1.13 (0.50)	0.915
I had much more energy than usual	1.30 (0.66)	1.28 (0.65)	1.27 (0.71)	1.32 (0.67)	1.40 (0.68)	1.28 (0.66)	0.934
I used an object (such as a towel) so I could avoid touching something directly	1.26 (0.74)	1.19 (0.54)	1.17 (0.63)	1.15 (0.51)	1.25 (0.70)	1.09 (0.46)	0.766
I was anxious about talking in public	1.32 (0.66)	1.47 (0.88)	1.23 (0.56)	1.29 (0.59)	1.19 (0.49)	1.20 (0.45)	0.252

Table 7 presents the B-IPF findings. The B-IPF is a self-reported questionnaire containing 80 items, measuring PTSD-related functional impairment in the past 30 days. There are seven functional domains evaluated, namely romantic relationships, family relationships, work, friendships and socializing, parenting, education, and self-care. There were no statistically significant changes over the study period.

**Table 7:** Brief Inventory of Psychosocial Functioning (B-IPF) scores.

Variable	Visit 1, N = 48	Visit 2, N = 48	Visit 3, N = 48	Visit 4, N = 48	Visit 5, N = 48	Visit 6, N = 48	Overall p-value
I had trouble taking care of myself	1.62 (1.42)	1.51 (1.20)	1.58 (1.30)	1.33 (1.06)	1.35 (1.00)	1.33 (1.00)	0.689
I was distressed or emotionally upset because of the difficulties I had taking care of myself	1.58 (1.35)	1.55 (1.40)	1.38 (0.84)	1.32 (0.91)	1.31 (0.66)	1.33 (0.72)	0.632
I had trouble in my romantic relationship with my spouse or partner	2.00 (1.70)	1.85 (1.44)	1.77 (1.25)	1.45 (0.93)	1.86 (1.33)	1.53 (0.86)	0.533
I was distressed or emotionally upset because of the difficulties I had in my romantic relationship	1.70 (1.31)	1.70 (1.40)	1.55 (1.15)	1.50 (1.01)	1.68 (1.16)	1.47 (0.82)	0.938
I had trouble with my family relationships.	1.74 (1.51)	1.62 (1.40)	1.41 (0.81)	1.58 (1.16)	1.45 (1.17)	1.31 (0.75)	0.606
I was distressed or emotionally upset because of the difficulties I had in my family relationships.	1.56 (1.14)	1.45 (1.11)	1.41 (0.84)	1.58 (1.10)	1.38 (0.94)	1.22 (0.42)	0.587
I had trouble at work	1.39 (0.72)	1.72 (1.36)	1.55 (1.15)	1.33 (0.71)	1.34 (0.94)	1.30 (0.70)	0.506
I was distressed or emotionally upset because of my difficulties at work	1.43 (0.77)	1.41 (1.24)	1.24 (0.44)	1.29 (0.64)	1.34 (0.86)	1.23 (0.43)	0.876
I had trouble with my friendships and socializing	1.29 (0.87)	1.30 (0.76)	1.31 (0.79)	1.44 (0.92)	1.11 (0.48)	1.24 (0.60)	0.433
I was distressed or emotionally upset because of the difficulties I had with my friendships and socializing	1.22 (0.77)	1.26 (0.61)	1.30 (0.73)	1.38 (0.84)	1.17 (0.64)	1.13 (0.40)	0.559
I had trouble in my relationship with my children	1.71 (1.52)	1.91 (1.51)	1.62 (1.58)	1.63 (1.50)	1.45 (1.41)	1.55 (1.50)	0.888
I was distressed or emotionally upset because of the difficulties I had in my relationship with my children	1.71 (1.37)	1.88 (1.66)	1.47 (1.08)	1.60 (1.35)	1.48 (1.18)	1.32 (1.09)	0.587
I had trouble at school	1.50 (1.07)	1.23 (0.83)	1.00 (0.00)	1.08 (0.28)	1.46 (1.66)	1.00 (0.00)	0.629
I was distressed or emotionally upset because of my difficulties at school	1.38 (0.74)	1.23 (0.83)	1.00 (0.00)	1.08 (0.28)	1.00 (0.00)	1.00 (0.00)	0.350

Table 8 contains the data from the PSQI. The PSQI is a self-reported questionnaire containing 19 items designed to measure sleep quality as well as sleep disturbances over a one-month period. The sleep scores or global score range from 0 to 21, with the higher total scores indicating worse sleep quality. The average global score of patients at baseline was 12.02 ± 4.66, and after the six-month study period, the average global score was 11.60 ± 5.45. The number of patients who reported waking up from pain decreased over the study interval and was trending toward statistical significance. Patients were able to report issues other than those on the PSQI, and some of the responses included “leg cramps,” “Just not tired,” “worried about family and friends,” “anxiety,” and “I use a CPAP machine.” Over the course of the six visits, patients who reported one of the above sleeping issues were having less of them at visit six when compared to visit one (p < 0.05).

**Table 8:** Pittsburgh Sleep Quality Index (PSQI) data.

Variable	Visit 1, N = 48	Visit 2, N = 48	Visit 3, N = 48	Visit 4, N = 48	Visit 5, N = 48	Visit 6, N = 48	Overall p-value
Cannot get to sleep within 30 minutes	2.08 (1.18)	2.21 (1.25)	2.02 (1.09)	1.75 (0.96)	1.88 (1.08)	1.85 (1.08)	0.355
Wake up in the middle of the night or early morning	3.19 (1.14)	3.35 (1.02)	3.13 (1.01)	2.93 (1.16)	3.00 (1.07)	2.77 (1.15)	0.137
Have to get up to use the bathroom	3.47 (0.97)	3.50 (0.88)	3.33 (1.04)	3.30 (0.98)	3.25 (1.06)	3.38 (1.04)	0.809
Cannot breathe comfortably	1.29 (0.68)	1.17 (0.56)	1.08 (0.35)	1.24 (0.74)	1.19 (0.45)	1.17 (0.52)	0.578
Cough or snore loudly	1.50 (0.85)	1.77 (0.93)	1.48 (0.85)	1.42 (0.85)	1.45 (0.85)	1.44 (0.80)	0.339
Feel too cold	1.38 (0.76)	1.35 (0.79)	1.46 (0.82)	1.44 (0.77)	1.44 (0.80)	1.52 (0.85)	0.929
Feel too hot	1.83 (1.15)	1.79 (1.11)	1.62 (1.01)	1.52 (0.90)	1.66 (0.92)	1.52 (0.92)	0.528
Had bad dreams	1.44 (0.77)	1.31 (0.59)	1.31 (0.59)	1.29 (0.58)	1.42 (0.74)	1.28 (0.66)	0.780
Have pain	2.96 (1.20)	2.96 (1.07)	2.48 (1.20)	2.58 (1.22)	2.58 (1.05)	2.43 (1.08)	0.080
How often during the past month have you had trouble sleeping because of this	3.20 (1.06)	2.67 (1.14)	2.43 (1.19)	2.03 (1.18)	2.19 (1.17)	2.22 (1.16)	0.006
During the past month, how would you rate your sleep quality overall	2.21 (0.72)	2.34 (0.73)	2.13 (0.65)	2.00 (0.71)	1.98 (0.58)	2.07 (0.75)	0.109
During the past month, how often have you taken medicine (prescribed or over the counter) to help you sleep?	1.81 (1.23)	1.72 (1.19)	1.74 (1.10)	1.71 (1.14)	1.64 (1.09)	1.91 (1.24)	0.915
During the past month, how often have you had trouble staying awake while driving eating meals, or engaging in social activity	1.20 (0.50)	1.26 (0.64)	1.18 (0.44)	1.09 (0.29)	1.07 (0.25)	1.11 (0.38)	0.279
During the past month, how much of a problem has it been for you to keep up enough enthusiasm to get things done	1.87 (0.83)	1.94 (0.79)	1.83 (0.74)	1.73 (0.81)	1.87 (0.83)	1.63 (0.72)	0.489
Loud snoring	2.03 (1.19)	1.83 (1.15)	1.97 (1.14)	1.72 (1.08)	1.83 (1.09)	1.74 (1.16)	0.862
Long pauses between breaths while asleep	1.48 (1.01)	1.18 (0.48)	1.19 (0.60)	1.12 (0.42)	1.29 (0.71)	1.25 (0.62)	0.407
Legs twitching or jerking while you sleep	1.79 (1.20)	1.66 (1.04)	1.73 (1.01)	1.56 (1.08)	1.80 (1.19)	1.70 (0.98)	0.960
Episodes of disorientation or confusion during sleep	1.10 (0.31)	1.10 (0.31)	1.10 (0.30)	1.16 (0.57)	1.03 (0.19)	1.06 (0.24)	0.814

Four linear regressions were run looking at the four pain scores as the main dependent outcomes (Table 9). In the first regression, which used pain levels right now as the dependent outcome, it was found that patients’ pain increases when they change position to get their back comfortable (0.125 (0.39-2.10) 0.005), the more irritable and bad-tempered patients are (1.25 (0.10-2.40) 0.035), the less patients eat (0.52 (0.02-1.02), 0.042), and increases in pain (0.50 (0.07-0.94) 0.024). However, the longer patients stand for leads to decrease in pain (-1.11 (-2.03-(-0.19)) 0.020). In the second regression that examined usual pain levels, increases in changing patients’ position (1.07 (0.34-1.80) 0.005), being more irritable and bad-tempered (1.45 (0.46-2.43) 0.005), and increases pain (0.48 (0.11-0.85) 0.012) remained consistent while staying significant. It was found that talking normal or quicker (-1.41 (-2.46-(-0.036)) 0.009) led to decreases in patients’ overall pain score. In the third regression that analyzed best pain score, it was found that males (-0.85 (-1.62-(-0.08)) 0.031) have decreases in their pain score. Sleeping less because of patients’ back (0.87 (0.03-1.71) 0.044) and being more irritable and bad-tempered (1.57 (0.59-2.55) 0.002) lead to increases in pain. The fourth and final regression examined the worst pain levels. It was found that changing patients’ position to get comfortable (1.69 (0.81-2.56) <0.001) and increases in pain (0.69 (0.24-1.13) 0.003) led to worse pain levels.

**Table 9:** Linear regression data.

Variable	Pain levels right now		Usual pain levels		Best pain levels		Worst pain levels	
	r (95% CI)	P-value	r (95% CI)	P-value	r (95% CI)	P-value	r (95% CI)	P-value
Age	0.03	0.305	0.02	0.352	0.03	0.227	0.01	0.643
Sex: male	-0.78	0.092	-0.71	0.081	-0.85	0.031	-0.71	0.134
I stay at home most of the time because of my back	0.59	0.0377	0.40	0.476	0.47	0.400	0.50	0.456
I change position frequently to try to get my back comfortable	0.125	0.005	1.07	0.005	0.36	0.336	1.69	<0.001
I walk more slowly than usual because of my back	0.82	0.103	0.25	0.561	0.81	0.058	0.36	0.480
Because of my back, I am not doing any jobs that I usually do around the house	0.49	0.306	0.46	0.271	0.39	0.336	0.25	0.615
Because of my back, I use a handrail to get upstairs	0.30	0.502	0.20	0.606	0.50	0.187	-0.51	0.266
Because of my back, I lie down to rest more often	-0.13	0.754	0.03	0.922	-0.26	0.447	-0.31	0.458
Because of my back, I have to hold on to something to get out of an easy chair	-0.17	0.736	-0.55	0.210	0.17	0.696	-0.02	0.967
I get dressed more slowly than usual because of my back	0.06	0.901	-0.16	0.685	0.10	0.788	0.35	0.457
I only stand up for short periods of time because of my back	-1.11	0.020	-0.43	0.294	-0.44	0.277	-0.17	0.732
Because of my back, I try not to bend or kneel down	0.49	0.223	-0.08	0.824	0.33	0.337	-0.60	0.146
I find it difficult to get out of a chair because of my back	0.09	0.884	0.70	0.164	0.36	0.467	0.10	0.871
I find it difficult to turn over in bed because of my back	-0.03	0.945	-0.07	0.857	-0.45	0.249	-0.61	0.194
I can only walk short distances because of my back pain	0.03	0.957	0.19	0.638	0.03	0.934	0.08	0.865
I sleep less well because of my back	0.70	0.166	0.10	0.820	0.87	0.044	0.23	0.662
I sit down for most of the day because of my back	0.34	0.510	0.30	0.493	0.33	0.441	0.89	0.093
I avoid heavy jobs around the house because of my back	-0.05	0.907	-0.10	0.800	-0.09	0.825	0.30	0.518
Because of back pain, I am more irritable and bad tempered with people than usual	1.25	0.035	1.45	0.005	1.57	0.002	0.06	0.921
Because of my back, I go upstairs more slowly than usual	-0.35	0.497	0.38	0.388	-0.22	0.609	0.17	0.750
I stay in bed most of the time because of my back	-1.47	0.397	-0.78	0.600	-0.11	0.941	-1.46	0.411
I did not have much of an appetite	0.52	0.042	0.43	0.051	0.15	0.491	0.38	0.145
I slept less than usual	-0.02	0.923	0.20	0.300	0.03	0.863	0.07	0.748
I had trouble falling asleep	0.04	0.854	0.01	0.970	-0.19	0.331	0.31	0.190
I talked more slowly than usual	-1.22	0.053	-1.41	0.009	-0.69	0.197	-0.42	0.508
Have pain	0.50	0.024	0.48	0.012	0.06	0.757	0.69	0.003
How often during the past month have you had trouble sleeping because of this	-0.34	0.094	0.15	0.981	-0.12	0.472	0.09	0.676
During the past month, how would you rate your sleep quality overall	-0.16	0.623	-0.22	0.451	0.27	0.339	-0.53	0.125

## Discussion

Pain management

This observational, prospective, open-label study saw patients with chronic pain related to neurogenic claudication or radicular leg symptoms due to lumbar spinal stenosis at one or more levels show improvement from baseline in pain outcomes following the use of hemp-derived CBD gel caps. Significant improvements were observed in patients’ usual levels of pain and their worst levels of pain. At baseline, the mean pain scores on an 11-point scale, 10 being the worst and 0 being no pain, were 6.02 ± 1.95 and 7.54 ± 2.07, respectively, indicating moderate to severe pain. The pain scores at visit six were 4.26 ± 2.41 and 5.75 ± 2.82, indicating mild to moderate pain. The nearly two-point decrease in pain scores throughout the study is indicative of a clinically significant improvement [15].

Medical cannabis has been shown to have pain-relieving properties in several different types of studies, including three short-term studies [16] and several long-term studies [17]. These studies analyzed the effects of plant-based cannabis in chronic pain patients and found significant improvements in their pain scales. While these results are in line with our findings, this study’s results are some of the first of their kind because only CBD was examined without THC. At the time this was being written, there were no other CBD studies of this kind published.

Medical cannabis has been observed to be relatively safe without significant adverse effects [18]. However, many of the adverse effects of medical cannabis stem from improper dosing of THC, suggesting that CBD may be a safer cannabinoid even at higher dosages [19]. While there is not much research surrounding CBD, one preclinical study found that CBD has an optimal dose, and below and above that dose, it was not as effective [20]. More research is needed to better understand the complex mechanisms, especially in human populations. As more states legalize cannabis, many precautionary measures need to be taken to help protect vulnerable populations from this largely unexplored drug. Moreover, medical cannabis must be used under the guidance and supervision of a physician, with regular follow-ups to optimize the dose and monitor tolerability and adverse events.

While pain scores were the primary outcome we evaluated in this study, many other data points were collected, looking at the impact pain has on mental and physical health. Because chronic pain negatively impacts the quality of life in many ways, when evaluating the effectiveness of pain treatments, it is vital to consider all aspects [21]. This study saw primarily beneficial results when examining the quality-of-life measures. Improvements were seen in patients’ ability to stand more due to less pain; they also changed positions less frequently with lower pain scores; they also experienced increased appetite with lower pain scores. Other statistically significant measures were that patients reported having less trouble falling asleep, sleeping more, and waking up less at night.

The four long-term, prospective, open-label studies mentioned above evaluated THC and CBD, i.e., Ware et al. [17], Haroutounian et al. [22], Bellnier et al. [23], and Safakish et al. [24], all found improvements in measures of quality of life after medical marijuana treatment. Each of these studies saw a more considerable increase in their quality-of-life measures. Given that this study only evaluated CBD without THC, it is possible that these four studies had better quality-of-life outcomes due to THC and CBD behaving in a dynamic and dose-dependent manner, modulating one another for more effective outcomes [25]. Another study that supports this study’s finding that CBD helps improve sleep is the study by Shannon et al. [26]. Nearly all patients were given CBD 25 mg/day in capsule form each morning. However, in this study, a handful of patients were given a higher dose of CBD, 50 mg/day to 175 mg/day. They observed that patients’ sleep scores improved and anxiety scores decreased [26].

There is no proven standard of care when looking at traditional spinal stenosis treatments. Zaina et al. found no difference in outcomes between surgical and conservative nonsurgical approaches [27]. Nonoperative treatment is widely used for the early stages of lumbar spinal stenosis but depends on several factors such as pain severity and the presence or absence of significant weakness. These more conservative treatments can entail medications such as nonsteroidal anti-inflammatory drugs, anti-epileptics, anti-depressants and/or opioids, physical therapy, or epidural steroid injections. While neither the surgical nor nonsurgical treatments of spinal stenosis have proven to be more effective in practice, many of these patients are still experiencing pain that impairs function. Adding CBD to their regimens might be another arrow in a physician’s quiver to help combat this debilitating diagnosis.

Safety profile

Today in the United States, cannabis is a Schedule I drug under the Federal Controlled Substance Act. However, the 2018 Farm Bill legalized hemp at the federal level by removing it from the list of controlled substances. Consequently, CBD from hemp is legal but cannot contain more than 0.3% THC. The many bioactive compounds in cannabis are known as cannabinoids, which bind to cannabinoid receptors (CB1 and CB2) on cell membranes [28]. These cannabinoid receptors typically bind endogenous cannabinoids (anandamide and 2-arachidonoylglycerol) [29]. CB1 receptors are mainly found in the central and peripheral nervous systems; CB2 receptors are primarily found on immune cells [28]. Two of the main exogenous cannabinoids are THC and CBD. THC has potentially intoxicating properties targeting the CB1 receptors; CBD, a nonintoxicating cannabinoid, appears not to bind directly to either CB1 or CB2 receptors and acts as an antagonist at CB1 receptors making CBD an appealing option for medical use [30]. This study did not report any adverse effects throughout the six visits from participants.

Generalizability of results

As previously indicated, each visit had fewer patients following up. This could have been from the observational study design and not directly handing patients the CBD gel caps. The dropout rate could have contributed to bias and limited the generalizability of results. Significant improvements were observed in the patient’s usual pain scores as well as their worst pain scores. These results serve as a stepping stone in the evidence showing a positive trend, suggesting the generalizability of the results.

Study limitations

There were several limitations associated with this study. This study was an open-label study, and there was no control group. Another limitation of the study was that each study visit saw people drop out of the study, which could have impacted our results. These participants dropping out may have been exacerbated by the lack of financial incentives to complete the surveys. The surveys themselves were a limitation of this study for two reasons, the first being that they were exceedingly long, leading to survey fatigue taking 30-45 minutes to complete. Second, because the surveys were long, it could have deterred patients from returning for future visits. Another limitation is selection/volunteer bias. Cannabis has a sizeable negative stigma surrounding it, and the patients open to trying CBD may have been more open to positive changes. The one major limitation of this study is that patients could receive other medical interventions such as physical therapy, injections, or other medications during the study, and it is not clear if the benefits seen in the outcome measures were due to CBD or other interventions. The hemp gel caps used in our study contained 15 mg of CBD, and the product label states that patients should use one capsule twice a day; two limitations surrounding these capsules are it is not known if 30 mg/day is the right dose for pain, and this study did not ask patients if they took the gel caps or how many a day. The last limitation is expectancy bias; the data were collected through self-reported surveys.

## Conclusions

This open-label, prospective, observational study found that treatment with hemp-derived CBD gel caps was associated with significant improvements in pain scores and several quality-of-life measures. Improvements in pain scores ultimately led to improvements in patients’ quality of life. This study did see fewer increases in quality-of-life measures than previous research involving both THC and CBD. However, the CBD gel caps were not associated with any adverse effects. Using CBD to help alleviate pain in spinal stenosis is supported by the evidence in this study. While first of its kind, this study supports the evidence that cannabis products can be a safe and effective treatment option for managing chronic pain.
